# Demographic and socioeconomic determinants of access to care: A subgroup disparity analysis using new equity-focused measurements

**DOI:** 10.1371/journal.pone.0290692

**Published:** 2023-11-16

**Authors:** Miao Qi, Henrique Santos, Paulo Pinheiro, Deborah L. McGuinness, Kristin P. Bennett

**Affiliations:** 1 Department of Computer Science, Rensselaer Polytechnic Institute, Troy, New York, United States of America; 2 Parcela Semântica Lda, Madeira, Portugal; 3 Department of Mathematical Sciences, Rensselaer Polytechnic Institute, Troy, New York, United States of America; Jinnah Sindh Medical University, PAKISTAN

## Abstract

Disparities in healthcare access and utilization associated with demographic and socioeconomic status hinder advancement of health equity. Thus, we designed a novel equity-focused approach to quantify variations of healthcare access/utilization from the expectation in national target populations. We additionally applied survey-weighted logistic regression models, to identify factors associated with usage of a particular type of health care. To facilitate generation of analysis datasets, we built an National Health and Nutrition Examination Survey (NHANES) knowledge graph to help automate source-level dynamic analyses across different survey years and subjects’ characteristics. We performed a cross-sectional subgroup disparity analysis of 2013-2018 NHANES on U.S. adults for receipt of diabetes treatments and vaccines against Hepatitis A (HAV), Hepatitis B (HBV), and Human Papilloma (HPV). Results show that in populations with hemoglobin A1c level ≥6%, patients with non-private insurance were less likely to receive newer and more beneficial antidiabetic medications; being Asian further exacerbated these disparities. For widely used drugs such as insulin, Asians experienced insignificant disparities in odds of prescription compared to White patients but received highly inadequate treatments with regard to their distribution in U.S. diabetic population. Vaccination rates were associated with some demographic/socioeconomic factors but not the others at different degrees for different diseases. For instance, while equity scores increase with rising education levels for HBV, they decrease with rising wealth levels for HPV. Among women vaccinated against HPV, minorities and poor communities usually received Cervarix while non-Hispanic White and higher-income groups received the more comprehensive Gardasil vaccine. Our study identified and quantified the impact of determinants of healthcare utilization for antidiabetic medications and vaccinations. Our new methods for semantics-aware disparity analysis of NHANES data could be readily generalized to other public health goals to support more rapid identification of disparities and development of policies, thus advancing health equity.

## Introduction

Ensuring fair and equitable health care access and utilization for people with the same health needs, regardless of their demographic and socioeconomic status, has become a primary goal of the Centers for Disease Control and Prevention (CDC) to eliminate health inequity and improve the U.S. public health and medical care systems [1]. However, studies have consistently shown that demographic characteristics (e.g., race, ethnicity, and gender) and socioeconomic characteristics (e.g., insurance type, education degree, and poverty level) are important factors contributing to the differences in health care access and utilization, which eventually lead to the dramatic life expectancy differences among subpopulations. For instance, the drug overdose mortality rate was 32.1% for individuals with Bachelor’s degree or more and 88.0% for those with lower education attainment in 2015-2019 [2]; diabetic patients from Black and lower median household income communities were 17% and 8% less likely to receive American Diabetes Association (ADA) guideline-recommended treatments such as sodium-glucose cotransporter type 2 inhibitors (SGLT2is) between 2015-2019 [3].

Essential medicines and vaccines, which “satisfy the priority health care needs of the population” [4], are the most frequently used healthcare services and have proven to effectively treat, manage, and prevent many diseases [5]. However, drug inaccessibility for certain cohorts of the population with specific diseases, such as type 2 diabetes mellitus (T2DM), has been associated with poor health outcomes [6], leaving patients at risk for serious medical issues. Similarly, the COVID-19 pandemic has again exposed widened inequities in vaccine access that led to preventable deaths [7]. In our study, we explored the utilization of antidiabetic medications and CDC-recommended vaccines as examples to demonstrate the application of our approach to evaluating equity of access in healthcare.

To identify and evaluate potential significant inequities in health care utilization, we develop an approach to decide whether a subgroup received sufficient health care services compared to their share in the target population of subjects who need the services. By definition, equality simply means to treat everyone the same exact way; equity, instead, means to treat people differently according to their needs [8]. In our study, equity aims to allocate health care resources/opportunities accordingly across subgroups based on their different circumstances/needs to achieve the same health outcomes; equality means to allocate the healthcare resources/opportunities evenly to different subgroups. In our previous work on equity of randomized clinical trials (RCTs), we developed a set of equity metrics with associated statistical tests that quantify if subgroups in a target population had disparate access to a clinical trial [9]. The associated significance test takes into account the disparities caused by the RCT size and estimation errors of ideal RCT access rate. This equity metric-based approach for RCT can be immediately generalized to the problem of identifying if subgroups in a target population exhibited disparate access to a specified health care service. For instance, this equity metric-based approach was generalized to show the disparities in vaccine usage for certain subgroups defined by race/ethnicity and socioeconomic status with respect to the U.S. population, such as the insufficient hepatitis A virus (HAV) vaccination usage in the non-Hispanic White population. In our study, we select one or more demographic and socioeconomic covariates (referred to as sensitive covariates) and then examine subgroups defined by one or more of these attributes. The proposed equity-focused approach identifies subgroups defined by multiple covariates that receive less or different health care service than expected within the target population.

We observe that our novel equity-focused method adds insights beyond those found by logistic regression [10]. As commonly done in the health domain, we use logistic regression to identify covariates associated with increased odds of specific healthcare utilization. Specifically, we use logistic regression to estimate if independent sensitive covariates have a significant relationship (quantified by odds ratios (ORs)) with the outcome treatment variable adjusted for confounding factors. Logistic regression uses no information about the target population requiring care and it typically doesn’t model interactions between the sensitive covariates. On the other hand, the proposed equity-focused approach delineates health care disparities received by subgroups defined over potentially multiple covariates compared to a specific target population.

We examined distributions of access to needed care, which include antidiabetic drugs and vaccines for HAV, hepatitis B virus (HBV), and human papillomavirus (HPV), across U.S. adults and studied associations with demographic features (e.g., gender and race/ethnicity) and socioeconomic determinants of health (e.g., education attainment, poverty income ratio (PIR), and insurance status) on patients’ reception of care.

For supporting these analysis requirements, we developed an approach based on Semantic Web technologies [11] to model and integrate National Health and Nutrition Examination Survey (NHANES) data as a Knowledge Graph. This approach formalizes NHANES survey knowledge that is present in the original datasets and online documentation as published by the CDC into machine-interpretable semantic data dictionaries [12] (SDDs), including codebooks. The constructed SDDs and related data were then used in a novel semantics-aware data integration framework to build the NHANES Knowledge Graph and to allow the generation of prepared datasets according to user selection of variables of interest. This approach helps automate data preparation, especially in settings like NHANES where it is common to combine data from multiple survey cycles, by representing the subject’s characteristics uniformly, regardless of dataset. The semantically-supported equity analysis can facilitate future equity analysis of services beyond those in this study.

Our study identified and quantified potential demographic and socioeconomic determinants of health care utilization for a range of healthcare problems involving diabetes treatment and vaccinations. For example, patients with non-private insurance might miss opportunities to be prescribed newer and more beneficial antidiabetic medications, and being non-Hispanic Asians further exacerbates disparities in types of prescribed antidiabetic drugs compared to other racial/ethnic groups. Additionally, our findings suggest that minorities have greater access to HAV and HBV vaccines, while non-Hispanic White populations are associated with a higher HPV vaccination rate. Specifically, minorities and poor communities tend to receive Cervarix, while non-Hispanic White and higher-income populations are more likely to receive Gardasil, which is a more comprehensive vaccine. The evidence suggests that analysis of equitable access and utilization of care should be performed routinely to support major public health goals and be considered in policies, thus advancing health equity and supporting better health for all. For example, the assessment methods proposed here can be used to examine how well clinical recommendations and guidelines are achieved in practice in different subgroups and appropriate public health interventions can be designed to address any problems identified.

Our main contributions include design and utilization of a health equity assessment methodology and infrastructure which:

- Introduces a methodology for identifying subgroups with disparate access to health care services.
- Determines the effects of various determinants of health on access to health care equity using new analysis and visualization methods.
- Develops a semantics-aware data integration framework to facilitate dynamic analyses across surveys for different research objectives.
- Applies the health equity assessment framework to evaluate utilization of antidiabetic drugs and vaccines.

In this paper, we first introduce the approaches that measure disparities of health care utilization between groups, including the novel subgroup disparity approach built upon our health equity framework [9] and the traditional logistic regression model. Then, we discuss details on selected demographic and socioeconomic determinants of health and on the method to estimate study and target populations from NHANES. Next, we applied the approaches to evaluate utilization of antidiabetic drugs, HAV, HBV, and HPV vaccines. Finally, we discuss the advantages and limitations of our equity-based approach and point out some potential directions of future work.

## Materials and methods

To identify the underlying determinants of inequitable health care access/utilization and to monitor health disparities in medication and vaccine utilization, we examined distributions of utilization of needed care, which include antidiabetic drugs, HAV, HBV, and HPV, across U.S. adults. We developed an approach based on the equity framework from our previous work [9] to study determined effects of demographic features (e.g., gender and race/ethnicity) and socioeconomic determinants of health (e.g. education attainment, poverty income ratio (PIR), and insurance status) on patients’ utilization of care in addition to logistic regression.

For medications, the outcome variable of interest is whether the participant was prescribed a drug from a specific Multum drug/ingredient therapeutic category [13]. This nested 3-level therapeutic classification scheme according to the Multum Lexicon is used to assign therapeutic categories for a drug or an ingredient of drug in NHANES. For vaccines, the outcome variable of interest is whether subjects received vaccine. We explore three types of vaccines available in NHANES on the population: HAV, HBV, and HPV. Two HPV vaccines, Cervarix and Gardasil, are additionally evaluated but constrained to people who received HPV vaccine.

### Subgroup disparity analysis

Our analyses on equitable health care utilization are based on two approaches: a new equity metric-based method and a logistic regression model frequently used for determinants of health. Logistic regression is a widely used statistical method for determining how a dependent variable is affected by one or more independent variables. The utilization analysis was performed separately for antidiabetic medications and vaccines using the following approaches.

#### Equity metric-based novel approach

Our equity metric-based approach is applied to decide whether the share of healthcare access and utilization across subgroups differentiated by demographic and socioeconomic factors of interest were proportionate to their share of the target population. To get clinically significant findings, we use subpopulation analysis to control for patient characteristics that differentiate drug prescribing of physicians by following treatment guidelines. For example, since hemoglobin A1c (HbA1c) level is clinically associated with the decision on prescribed antidiabetic drug class, we analyzed the effect of demographic and socioeconomic factors within subpopulations that have similar HbA1c levels. Multivariable conditions, such as both HbA1c level and the Charlson Comorbidity Index (CCI) level [14], can be applied to obtain a subpopulation-level equity heatmap for each subgroup defined over the conditioned attributes. This method quantifies disparities, providing opportunities to monitor and improve health equity improvement.

In this approach, a statistical analysis is performed to test if the disparities between the subgroups’ observed and target utilization rates of health care services are significant. If the p-value is smaller than the significance threshold 0.05, then we define that the subgroup received adequate service they needed; if p-value ≥0.05, then an equity metric [9] is applied to calculate a score that determines whether the utilization rate of health care service is inappropriate for the population. For each equity metric, an upper threshold *τ*_*u*_ and a lower threshold *τ*_*l*_ of metric values are defined to categorize metric scores into different equity levels. In our evaluation, the Log Disparity metric [9],

$$\begin{array}{c} \text{Log}\;\;\text{Disparity}\;\text{=}\;\text{log}\;\frac{odds\;of\;observed\;access\;to\;care}{odds\;of\;target\;access\;to\;care} \end{array}$$ (1)

is used. The threshold is *τ*_log disparity_ = −*log*(1 − *τ*_*rule*_), where *τ*_*rule*_ ∈ [0, 1]. We used the same standard set of *τ* values utilized in prior studies of randomized clinical trials representativeness and equitable design [9, 15]. By following the 80% rule [16], our *τ*_*l*_ = −*log*(0.8). The *τ*_*u*_ is user-defined and was selected to be −*log*(0.6) in our experiment.

The color description of equity evaluation heatmaps is available in Table 1. The severity of inequity is mapped to color to help us visualize where disadvantaged subpopulations are, discover social determinants of health care utilization, or identify communities that need immediate intervention.

**Table 1:** Color description for heatmap.

Color	Description	Equity Score
Red	Absent	*
Orange	Highly Inadequate	< -*τ*_*u*_
Light orange	Inadequate	[-*τ*_*u*_,-*τ*_*l*_)
Teal	Adequate	[-*τ*_*l*_,*τ*_*l*_) or p > 0.05
Light blue	Abundant	[*τ*_*l*_,*τ*_*u*_)
Blue	Highly Abundant	≥ *τ*_*u*_

Cells are shaded in corresponding colors used in the visualizations.

#### Multivariate logistic regression model

We also used the multivariate logistic regression model, which is a popular and widely used association analysis method in health domain [17–22], to determine effects of demographic features and socioeconomic determinants of health on the access and utilization of health care. Since the multivariate logistic regression can explain the simultaneous effect of covariates on a dichotomous outcome, it is suitable for our study that involves various covariates by taking into account the correlations between different variables of interest.

We construct the model as a function of race/ethnicity, age, gender, educational attainment, insurance type, poverty level, comorbidity severity based on CCI, and HbA1c condition. The OR is the odds of drug/vaccination access and utilization of a subgroup divided by the odds of the same healthcare source access and utilization in a reference group. Reference groups are non-Hispanic White, male, and private insurance for unordered categorical variables; reference groups are the lowest level of ordered ones (e.g., CCI = 0, HbA1c condition < 6%). This method controls for the influence of approved time for usage by FDA across medications through age adjustment.

To apply logistic regression, we use the R package “svydiags” [23] to check the assumptions considering the complex survey design of NHANES. The assumptions of logistic regression are satisfied except that extreme outliers exist in our data. One limitation of logistic regression is that these assumptions are not always satisfied. In the experiment, extreme outliers represent patients who have special characteristics related to their access to/utilization of a health care service, which fail to conform with the rest of patients in the fitted logistic regression model. These extreme outliers can influence the estimates of the effect of a covariate on the outcome.

### Data source and study population

In the study, we analyzed the NHANES demographic, socioeconomic, diabetes, and vaccination data for the 2013-2018 survey cycles [24]. Participants from 2013 to 2018 survey cycles without missing data in the variables for analysis were included. All the NHANES programs were approved by the National Center for Health Statistics (NCHS) Ethics Review Board and the informed consent was signed by all subjects. The NHANES data used in the manuscript are de-identified and remain anonymous during the analysis. In the data released by the NCHS, all information that could identify the subject has been removed. The NCHS strictly follows the federal laws to keep participant information confidential [25]. Therefore, no ethical approval for this secondary research was required. To make estimates of the health-related statistics obtained as if the whole U.S. civilian non-institutionalized population have been surveyed, NHANES applied a stratified four-stage sampling designed to assign a sample weight to each sample person. The R survey package [26] is designed to automatically take into account these sample weights for each data point to perform correct analysis adjustments. By using appropriate weights based on the survey cycles and the subjects’ characteristics explored in our analysis, we could then estimate demographics and healthcare usage rates for a nationally representative population.

When we first analyzed NHANES, we used the traditional approach of having scientists conduct the process of inspecting the data, reading the data dictionaries, and confronting their interpretation of the data against online documentation and the data understanding developed by other scientists. By contrasting one interpretation against other interpretations, we collectively acquired a clarification on the exact meaning of the raw data that is often lost after similar analyses are done. This time, however, we have preserved this data understanding by translating human-level data dictionaries and codebooks into machine-level semantic data dictionaries and codebooks. Further, we have used these machine-level documents along with raw data and a novel semantic data integration infrastructure [27] to build an NHANES knowledge graph [28] that is publicly available at http://nhanes.eci.ufmg.br:9000/hadatac.

With the knowledge graph and infrastructure in place, we have repeated our original analysis using the traditional approach and used it to compare against the results produced from datasets automatically generated (prepared) from the infrastructure. The obtained results were identical, indicating that future analysis of NHANES data can have their expensive data preparation work expedited through the use of our semantic infrastructure.

#### Semantic-aware data integration approach

In NHANES, we experience common disconnects between data and knowledge that had to be addressed. For instance, data dictionaries (DDs) used to support data understanding by humans cannot be easily leveraged by machines. The DDs include natural language descriptions of the variables composing a dataset, as well as codebooks for select variables where codes are used instead of direct values. From the DDs we have created SDDs [12] where an identified associated entity, attributes, unit, set of code book values, time, space, and provenance properties are used to formalize the knowledge related to each variable.

During the creation of SDDs, we were thoughtfully when referencing appropriate terminology from existing established vocabularies and ontologies, employing ontology engineering best practices [29]. When specific NHANES terms were not found in reliable sources, we employed ontology engineering best practices to extend existing terminology, compiling these new terms in the NHANES ontology (https://github.com/tetherless-world/nhanes-hadatac). The NHANES ontology contains additional terminology for representing NHANES-specific entities, their attributes and roles, and codebook entries. The introduction of the NHANES ontology, alongside the reuse of established terminology, facilitates the integration of the NHANES data with data different sources, as well as within NHANES itself, such as during survey cycle aggregation. For example, in cases where different codebook entries for different variables describe the same value (e.g. “high-school degree’’ for both the subject and household reference person’s level of education) can be integrated using the ontology by utilizing the same resource. Similarly, different variables (usually from different survey cycles) may describe a similar entities’ attribute, in which the use of a single ontology resource describing such attribute helps the combination of these variables (e.g. different variables to characterize country of birth, depending on the survey cycle).

The semantic enhancements benefit data interpretation. For example, some of the codebooks provide categorical codes that group a range of possible values into a single code. One example is the “education level” variables which have codes that include “less than 9th grade” and “9-11th grade” for survey participants, and “less than high school degree” for household reference persons. In this case, the more discrete ranges in the survey participants’ codebook were defined as subclasses of the broader definition in the household reference persons’ codebook in the NHANES ontology. This modeling allows the inference that persons with the education level of “less than high school degree” can be grouped together with persons education level of “less than 9th grade”.

**Fig 1:**
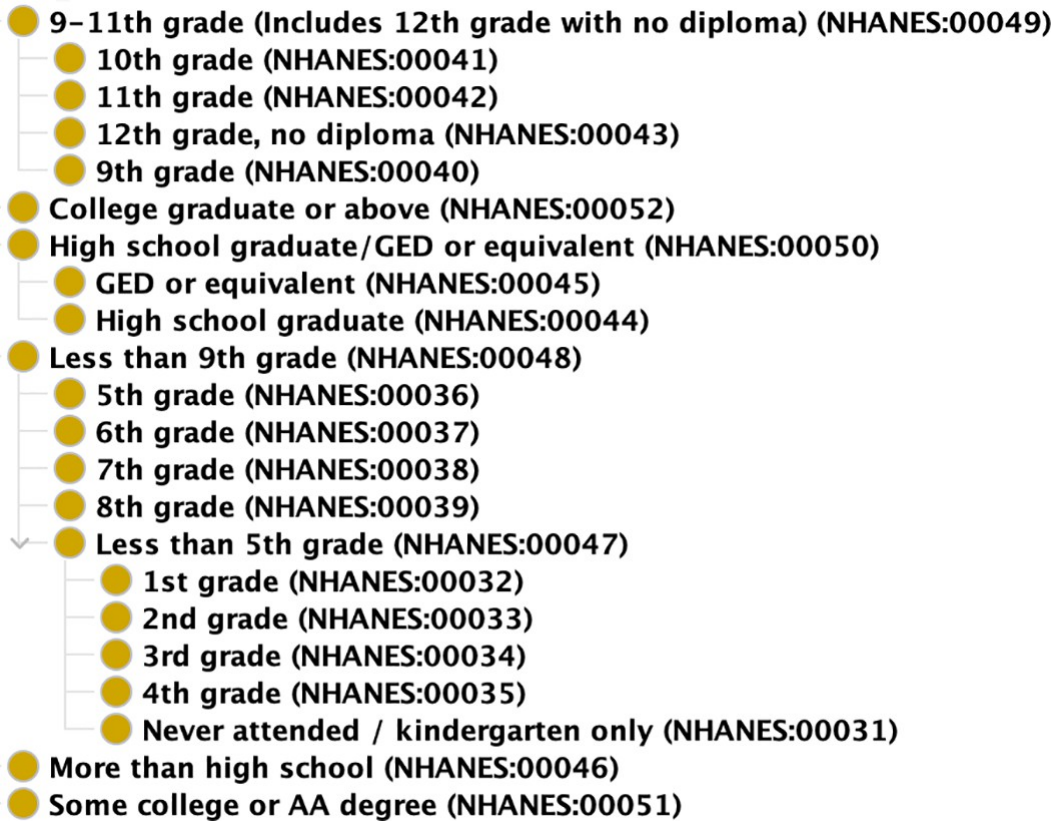
NHANES ontology. Part of our NHANES Ontology showcasing the harmonization of the several education levels across datasets.

Fig 1 shows part of our ontology covering some of the education classes mentioned above. This principle was applied to other variables where codebooks could be interpreted to identify similar relationships, and the results were incorporated into our NHANES ontology.

The produced SDDs, the NHANES ontology, and original NHANES datasets were used in conjunction to construct the NHANES Knowledge Graph. For the complete knowledge graph construction process, we refer the reader to [30].

#### Vaccination

We use the three types of vaccines (i.e., HAV, HBV, and HPV) available in NHANES immunization documents [31–33] to identify and evaluate potential inequitable access and utilization of immunization services experienced by some subgroups.

The demographic characteristics used in our analysis include self-reported age, gender (male, female), and race/ethnicity (non-Hispanic White, non-Hispanic Black, non-Hispanic Asian, Hispanic, or other/unknown). The socioeconomic characteristics include education level, insurance type, and the ratio of family income to poverty. The categorization of these variables follows. Education levels available from NHANES include less than 9th grade, 9-11th grade (includes 12th grade with no diploma), high school graduate/GED or equivalent, some college or AA degree, and college graduate or above. Health insurance status was classified as private insurance, Medicare, Medicaid, and other non-private insurance. The ratio of family income to poverty were categorized into poor (< 1), near poor (1-1.9), middle income (2-3.9), and higher income (≥ 4).

Data preparation issues are also present in these socioeconomic determinants. For instance, the Insurance datasets contain several variables used to fully characterize insurance coverage. Each variable contains the participation status of the survey participant in one specific type of insurance (such as Medicaid, Medicare, Private insurance, etc.). However, we understand insurance coverage as not the value of a single variable but the combination of several variables insurance-related variables, all contributing to the insurance coverage attribute of the participant. For example, we can only infer if a person does not have insurance coverage if all variables contain the information of not being covered. With the use of our NHANES semantic infrastructure, insurance coverage is available as a multivalued variable.

For HAV/HBV vaccination, we included subjects over age 20 and not pregnant based on the urine pregnancy test result; for HPV vaccination, only participants aged between 20 and 59 were analyzed due to the NHANES design [31].

#### Antidiabetic medication

We use antidiabetic medications as an example to identify and evaluate potential over-/under- prescribing or over-/under-use of certain types of medications to treat chronic conditions experienced by some populations.

The same demographic (i.e., age, gender, and race/ethnicity) and socioeconomic (i.e., education level, insurance type, and ratio of family income to poverty) factors for vaccinations are used for T2DM. However, the age categorization is updated to 20-45, 46-64, ≥ 65 according to disease domain knowledge from CDC [34].

For antidiabetic drug analysis, we included subjects with known T2DM, aged over 20, and nonpregnant. Additionally, we focused on T2DM treatments by excluding medications used for type 1 diabetes and for prevention. It was important for the semantic infrastructure to differentiate between the use of drugs to treat disease and to prevent disease, which is convoluted in the raw data since this is a distinction that was introduced later in the study. In our modeling, drug usages for treatment and prevention are separate variables.

To explore the effect of demographic features and socioeconomic determinants of health on patients’ access to ADA recommended T2DM treatments [35], we included 60 antidiabetic drugs available in NHANES and grouped them based on the Multum Lexicon therapeutic classification scheme [13]. The Multum Lexicon category system is used for drug name coding and therapeutic categories mapping. It is one of the coding methods for medications and used by the NHANES. The 10 categories we used include meglitinides, SGLT2is, sulfonylureas (SUs), biguanides, dipeptidyl peptidase IV inhibitors (DPP-4is), insulin, thiazolidinediones (TZDs), glucagon like peptide-1 receptor agonists (GLP-1RAs), *α*-glucosidase inhibitors (AGIs), and antidiabetic combinations.

According to the 2022 ADA guideline [35], treatment recommendation for adults with T2DM depends on comorbidities. So, 15 common comorbidities were considered in our analysis: hypertension, asthma, arthritis, gout, congestive heart failure, coronary heart disease, heart attack, stroke, emphysema, chronic bronchitis, cancer, liver disease, COPD (chronic obstructive pulmonary disease), kidney disease, and diabetic retinopathy. The CCI scores [14] of participants were calculated as an indicator of severity of comorbidity and were categorized into four levels: none (CCI score = 0); mild (CCI scores of 1–2); moderate (CCI scores of 3–4); and severe (with CCI scores ≥ 5) [36]. Another factor that influences antidiabetic medication prescription is Hemoglobin A1C (HbA1c), which was categorized into < 6%, 6%–7%, 7%–9%, and ≥ 9% [37].

## Results

The equity analysis on different types of diabetic medications and vaccines suggest the existence of different determinants of healthcare access and utilization for resources/services catering to different health needs, as discussed later in the text.

### Impact of demographic and socioeconomic factors on vaccination utilization

Table 2 displays results from our logistic regression model of vaccination coverage for the independent variables: racial/ethnic subgroups (reference: non-Hispanic White), different income subgroups (reference: poor population), subgroups of different insurance types (reference: private insurance), and subgroups with different educational attainments (reference: less than 9th grade). Minorities were more likely to be vaccinated against HAV/HBV compared to non-Hispanic White subjects. For instance, non-Hispanic Asian subjects had a 17% increase in the odds of getting HAV vaccine and a 10% increase in the odds of getting HBV vaccine compared to non-Hispanic White subjects. Similar analyses were performed for other factors of interest, revealing different levels of disparities. For example, the higher-income population had a 4% increase in the odds of getting HAV vaccination compared to the poor subjects; adults with an education level of 9-11th grade had a 8% increase in the odds of getting HAV and a 14% increase in the odds of getting HBV vaccinations compared to the subjects with less than a 9th grade education.

**Table 2:** Associations between U.S. population groups and vaccination by race/ethnicity, poverty level, insurance type, and education level.

Vaccine	HAV	HBV	HPV
NH Black	1.09*(1.03, 1.16)	1.05 (1.00, 1.10)	0.99 (0.94, 1.04)
NH Asian	1.17*(1.08, 1.26)	1.10*(1.04, 1.17)	0.94*(0.89, 0.99)
Hispanic	1.13*(1.08,1.18)	1.03 (0.97, 1.08)	0.99 (0.94, 1.05)
Near poor	1.01 (0.95, 1.06)	1.00 (0.94, 1.06)	1.02 (0.95, 1.08)
Middle income	0.99 (0.95, 1.04)	0.99 (0.94, 1.04)	0.98 (0.94, 1.02)
Higher income	1.04*(1.01, 1.08)	1.03 (0.99, 1.07)	0.98 (0.93, 1.04)
Medicaid	1.09*(1.01, 1.17)	1.03 (0.95, 1.12)	1.02 (0.93, 1.11)
Medicare	1.01 (0.94, 1.09)	0.99 (0.90, 1.09)	1.06 (0.93, 1.22)
Other insurance	1.01 (0.92, 1.10)	1.03 (0.93, 1.14)	1.02 (0.96, 1.09)
9-11th grade	1.08*(1.02, 1.15)	1.14*(1.06, 1.23)	1.02 (0.96, 1.08)
High school graduate	1.06 (1.00, 1.12)	1.04 (0.97, 1.10)	1.01 (0.96, 1.07)
Some college or AA degree	1.01 (0.96, 1.05)	0.98 (0.94, 1.03)	1.00 (0.95, 1.06)
College graduate or above	0.99 (0.94, 1.05)	0.98 (0.94, 1.02)	0.97 (0.92, 1.01)

Reference groups: race/ethnicity = non-Hispanic White; poverty level = poor; insurance type = private insurance; education level = less than 9th grade.

* indicates statistically significant disparity with p ≤ 0.05.

**Fig 2:**
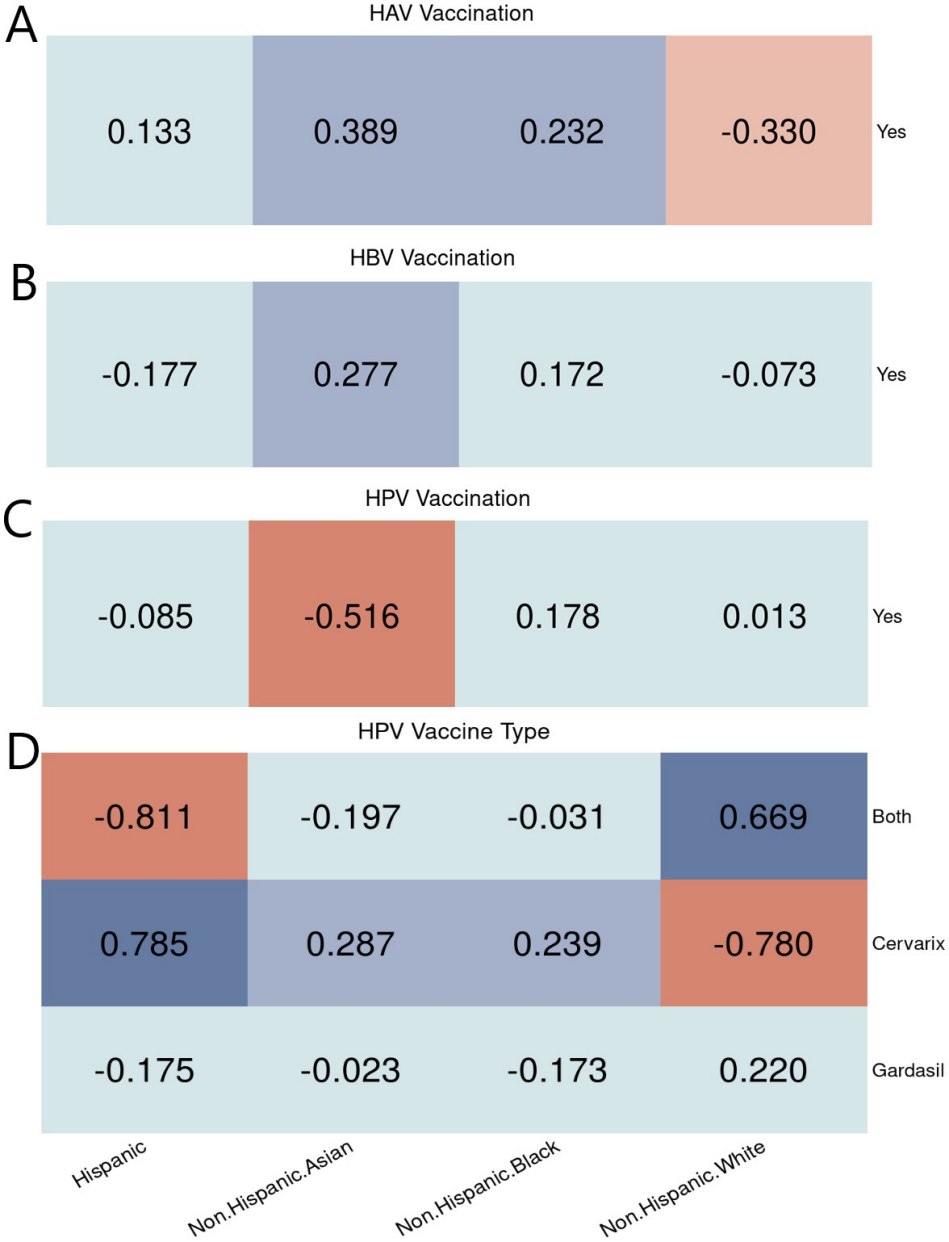
Racial/ethnic equity evaluation on vaccination. Equity evaluation heatmaps of racial/ethnic disparities on different cases. (A) HAV vaccination. (B) HBV vaccination. (C) HPV vaccination. (D) HPV vaccine types used among people who got the vaccine.

Equity-focused results for the same independent and outcome variables as for the logistic regression are shown as heatmaps. Heatmaps provide an immediate and comprehensive visual summary on how the access and utilization of health care services vary across different subgroups of interest. This visual representation of numeric equity-focused results assists the readers effectively undercover hidden trends and translate them into a better understanding of the equity issue in health care. In general, the trends are the same as those observed in Table 2 but it further detects the potential inequities in the reference population used for the logistic regression. Fig 2 shows that the Hispanic population received adequate vaccination coverage for all three types. The non-Hispanic Asian population had higher HAV and HBV vaccination coverage but lower HPV vaccination coverage. The non-Hispanic Black population had higher HAV vaccination coverage. In contrast, the non-Hispanic White population did not receive sufficient HAV vaccination. Furthermore, Fig 2D indicates that minorities tended to receive Cervarix more frequently than the Gardasil vaccine, which is designed to treat more types of infections.

**Fig 3:**
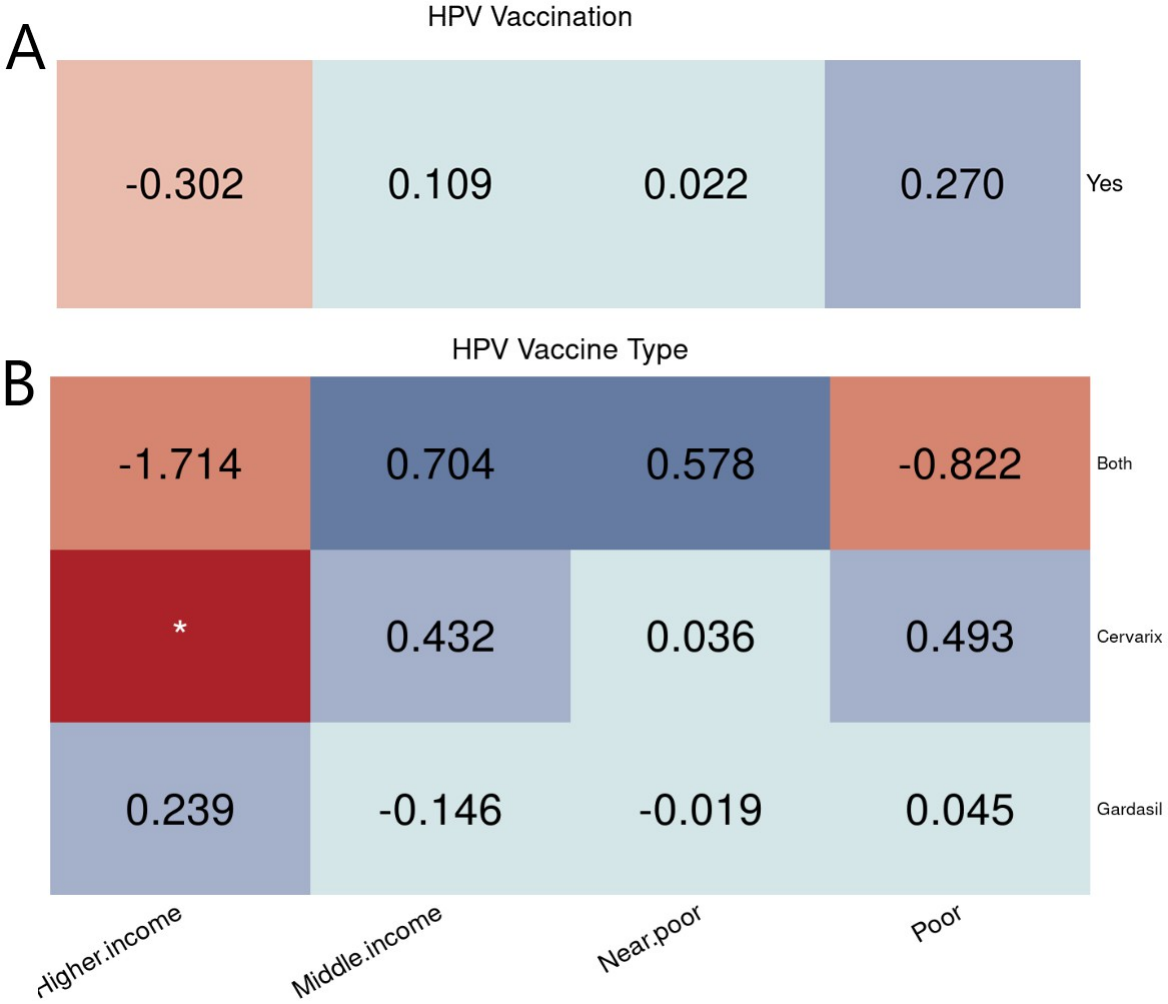
Poverty level equity evaluation on vaccination. Equity evaluation heatmaps of disparities by poverty level on different cases. (A) HPV vaccination. (B) HPV vaccine types used among people who got the vaccine.

The equity results found no significant relationships between poverty level and HAV or HBV vaccinations. For HPV, Fig 3 shows that poor people were more likely to receive HPV vaccination, while the higher-income population did not achieve the target rate. Additionally, people with higher income were more likely to receive Gardasil, while other groups received more Cervarix. Regarding the logistic regression, the higher-income subgroup had an increased adjusted odds of receiving HAV compared to the poor subgroup.

**Fig 4:**
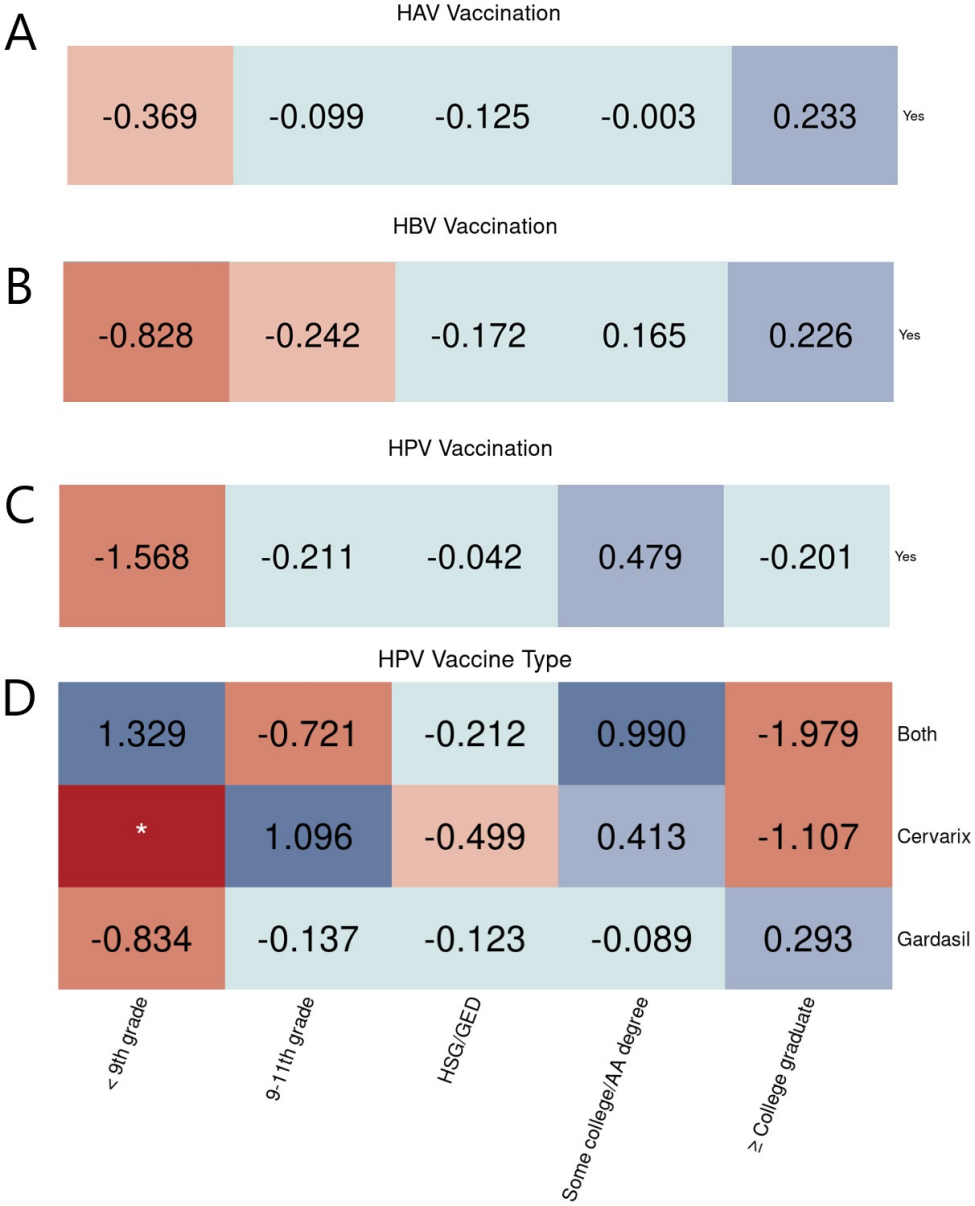
Education level equity evaluation on vaccination. Equity evaluation heatmaps of disparities by education level on different cases. (A) HAV vaccination. (B) HBV vaccination. (C) HPV vaccination. (D) HPV vaccine types used among people who got the vaccine.

Analyses based on other demographic and socioeconomic factors were performed. For instance, as shown in Fig 4, people who did not finish 9th grade tended to receive fewer vaccinations, while those with higher education attainment received a sufficient or sometimes even a larger share of vaccinations.

### Impact of demographic and socioeconomic factors on antidiabetic drug utilization

Tables 3–6 display findings from the logistic regression model on antidiabetic medication utilization, comparing the same subgroups as described for vaccines. For example, for the widely used drug, such as biguanides, non-Hispanic Black patients had a 10% decrease in the odds of prescribing rate compared to the reference group. Non-Hispanic Asians had a 6% decrease and a 4% decrease in the odds of prescribing rates for GLP-1RAs and TZDs, but a 14% increase in the odds of prescribing rates for SUs compared to the non-Hispanic White patients. Near-poor population had a 2% increase in the odds of prescribing rate for SGLT-2is compared to the poor population. No significant disparities were discovered between medication utilization and insurance types for all medication classes. Some education-level-based disparities also existed, such as a 9% decrease in the odds of prescribing rate for biguanides among the population with a college degree and higher education compared to those with less than a 9th grade education.

**Table 3:** Associations between U.S. population groups and diabetes drug use by race/ethnicity.

Medication Class	NH Black	NH Asian	Hispanic
AGIs	1.00 (1.00,1.01)	1.00 (0.99,1.00)	1.00 (0.99,1.01)
Biguanides	0.90*(0.81,0.99)	1.02 (0.89,1.17)	1.00 (0.91,1.11)
DPP-4is	0.97 (0.92,1.03)	1.04 (0.96,1.13)	1.02 (0.95,1.10)
GLP-1RAs	0.99 (0.94,1.04)	0.94*(0.88,1.00)	0.97 (0.93,1.01)
Insulin	1.03 (0.91,1.16)	0.89 (0.79,1.01)	0.95 (0.87,1.05)
Meglitinides	1.00 (0.99,1.02)	1.00 (0.99,1.02)	1.00 (0.99,1.01)
SGLT-2is	1.02 (0.98,1.06)	1.01 (0.96,1.06)	1.00 (0.97,1.03)
SUs	1.01 (0.91,1.11)	1.14*(1.02,1.27)	0.98 (0.89,1.08)
TZDs	1.01 (0.97,1.06)	0.96*(0.93,1.00)	1.02 (0.98,1.06)
Combinations	1.02 (0.97,1.07)	1.01 (0.95,1.06)	1.02 (0.97,1.08)
None	0.99 (0.94,1.05)	1.00 (0.95,1.05)	1.03 (0.95,1.11)

Reference group: non-Hispanic White. NH = non-Hispanic.

* indicates statistically significant disparity with p ≤ 0.05.

**Table 4:** Associations between U.S. population groups and diabetes drug use by poverty level.

Medication Class	Near poor	Middle income	Higher income
AGIs	1.00 (0.99,1.00)	1.00 (0.99,1.01)	1.01 (0.99,1.03)
Biguanides	1.02 (0.88,1.17)	0.96 (0.89,1.04)	0.98 (0.91,1.06)
DPP-4is	1.04 (0.97,1.12)	1.01 (0.96,1.07)	1.00 (0.94,1.06)
GLP-1RAs	1.04 (0.99,1.10)	1.03 (0.98,1.08)	1.00 (0.95,1.05)
Insulin	0.92 (0.82,1.03)	1.03 (0.95,1.11)	1.02 (0.95,1.09)
Meglitinides	1.00 (0.99,1.01)	1.00 (0.99,1.01)	1.00 (0.99,1.00)
SGLT-2is	1.02*(1.00,1.04)	1.01 (0.99,1.03)	1.00 (0.99,1.01)
SUs	1.03 (0.94,1.12)	0.97 (0.91,1.05)	0.96 (0.89,1.04)
TZDs	1.02 (0.97,1.08)	1.00 (0.96,1.04)	0.98 (0.95,1.01)
Combinations	0.98 (0.94,1.03)	1.02 (0.98,1.05)	0.99 (0.97,1.01)
None	0.99 (0.94,1.06)	0.98 (0.95,1.01)	1.02 (0.98,1.06)

Reference group: poverty level = poor.

* indicates statistically significant disparity with p ≤ 0.05.

**Table 5:** Associations between U.S. population groups and diabetes drug use by insurance type.

Medication Class	Medicaid	Medicare	Other insurance
AGIs	1.01 (0.99,1.02)	1.00 (0.99,1.02)	0.99 (0.98,1.01)
Biguanides	0.94 (0.79,1.12)	0.91 (0.79,1.06)	0.99 (0.85,1.14)
DPP-4is	0.99 (0.93,1.06)	1.02 (0.95,1.08)	1.02 (0.92,1.14)
GLP-1RAs	1.01 (0.89,1.13)	1.00 (0.93,1.09)	0.95 (0.90,1.00)
Insulin	1.07 (0.93,1.23)	1.06 (0.96,1.17)	0.96 (0.81,1.14)
Meglitinides	1.01 (0.99,1.02)	1.00 (0.99,1.01)	1.00 (1.00,1.00)
SGLT-2is	1.01 (0.97,1.05)	1.00 (0.98,1.01)	0.99 (0.95,1.03)
SUs	0.94 (0.81,1.10)	1.01 (0.92,1.10)	0.91 (0.79,1.05)
TZDs	0.98 (0.95,1.01)	1.00 (0.95,1.05)	0.98 (0.95,1.02)
Combinations	1.01 (0.95,1.07)	1.02 (0.98,1.08)	0.99 (0.95,1.04)
None	1.04 (0.94,1.14)	1.01 (0.94,1.09)	1.01 (0.91,1.11)

Reference group: insurance type = private insurance.

* indicates statistically significant disparity with p ≤ 0.05.

**Table 6:** Associations between U.S. population groups and diabetes drug use by education level.

Medication Class	9-11th grade	High school graduate	Some college / AA degree	College graduate or above
AGIs	1.00(0.98,1.01)	1.00(0.99,1.01)	1.00(0.99,1.01)	0.99(0.98,1.01)
Biguanides	1.07(0.93,1.22)	1.06(0.96,1.17)	1.03(0.94,1.13)	0.91*(0.82,1.00)
DPP-4is	0.97(0.89,1.06)	1.01(0.96,1.08)	1.02(0.96,1.08)	1.00(0.92,1.08)
GLP-1RAs	1.04(0.98,1.10)	1.06*(1.00,1.12)	1.03(0.96,1.09)	1.02(0.98,1.07)
Insulin	1.02(0.94,1.11)	0.98(0.90,1.07)	1.03(0.96,1.11)	1.02(0.96,1.10)
Meglitinides	0.99(0.98,1.00)	1.00(0.99,1.01)	1.00(0.99,1.01)	1.00(0.99,1.01)
SGLT-2is	0.99(0.96,1.02)	1.03(0.99,1.07)	1.00(0.97,1.03)	1.00(0.98,1.02)
SUs	0.94(0.85,1.04)	0.92(0.85,1.00)	1.11*(1.03,1.18)	1.03(0.94,1.13)
TZDs	0.98(0.91,1.06)	1.00(0.95,1.06)	1.03(0.99,1.08)	1.01(0.95,1.07)
Combinations	1.01(0.95,1.07)	1.03(0.98,1.09)	0.99(0.96,1.03)	1.01(0.98,1.04)
None	0.99(0.95,1.03)	0.98(0.94,1.03)	0.98(0.95,1.01)	1.04(1.00,1.09)

Reference group: education level = less than 9th grade.

* indicates statistically significant disparity with p ≤ 0.05.

**Fig 5:**
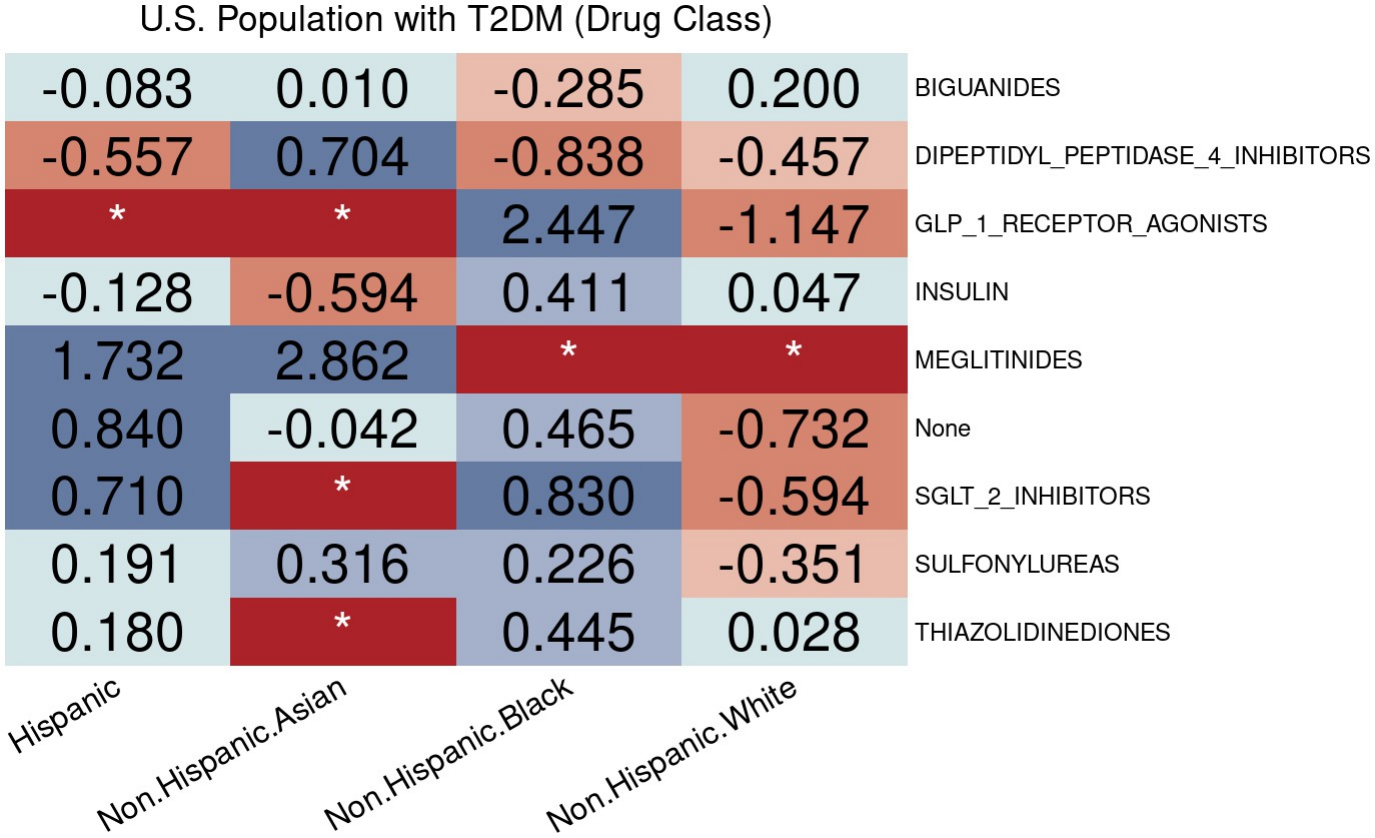
Disparities of hyperglycemic medication utilization by race/ethnicity.

Findings based on the log disparity metric are displayed in Fig 5. Here, the medications are categorized into the Multum Lexicon therapeutic categories based on ingredients, which further categorize the antidiabetic combinations into the other 9 categories. This analysis takes into account the disparities due to the unspecific information in the combinations. This analysis reveals potential disparities in the utilization of antidiabetic medications. For instance, GLP-1RAs seem to be highly overprescribed to non-Hispanic Black patients and insulins may be highly underprescribed to non-Hispanic Asian patients.

**Fig 6:**
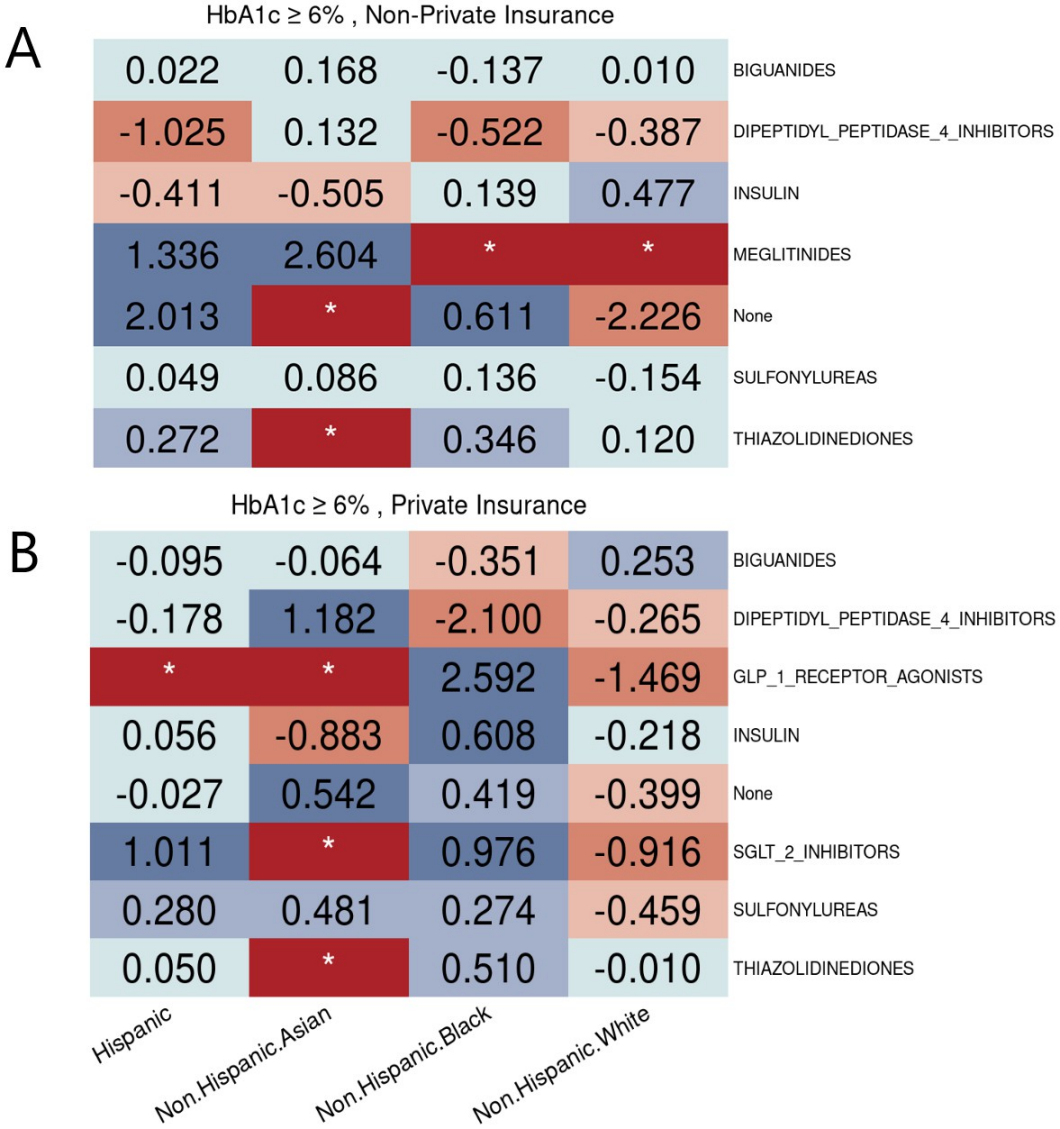
Racial/ethnic equity evaluation on hyperglycemic medication utilization. Racial/Ethnic disparities of hyperglycemic medication utilization among diabetic population with HbA1c ≥ 6% who have different types of insurance. (A) People with non-private insurance. (B) People with private insurance.

According to Fig 5, disparities are observed in non-Hispanic Black patients for the utilization of biguanides among U.S. adults with diagnosed diabetes. When exploring subpopulations with HbA1c ≥ 6% in Fig 6, we found that the disparities might be due to insurance types. For example, non-Hispanic Black patients with private insurance were less likely to be prescribed biguanides. This indicates that certain factors only have effects on specific subgroups conditioned on disease-specific determinants of prescribing decisions. Fig 6 also shows that patients with private insurance had access to newer antidiabetic drugs, such as SGLT2is and GLP-1RAs, recommended by the ADA guideline [35], while their counterparts with non-private insurance had limited opportunities to receive these new effective treatments.

**Fig 7:**
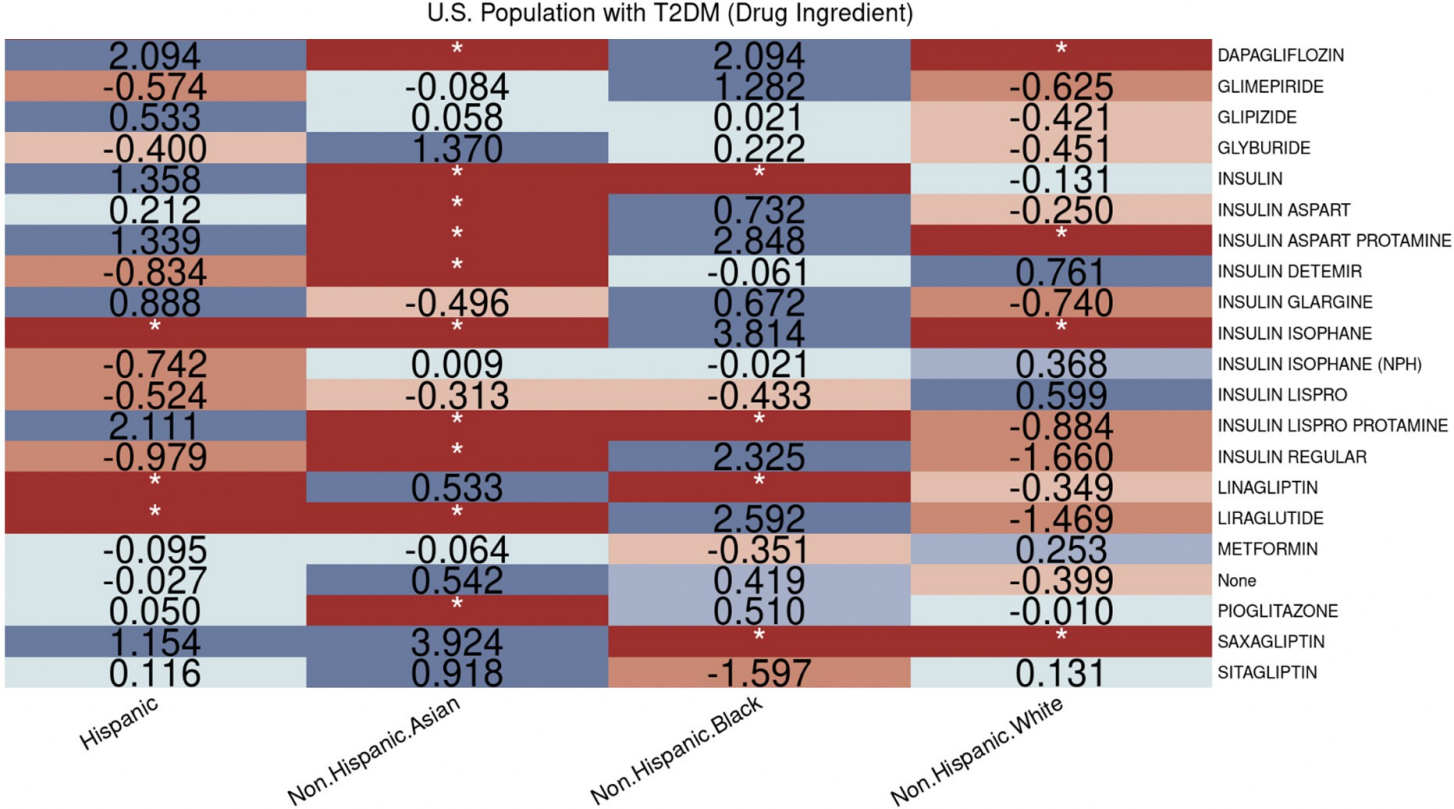
Disparities of ingredient utilization in hyperglycemic medications by race/ethnicity.

Additionally, we analyzed the access and utilization of antidiabetic ingredients, providing a deeper understanding of the root of disparities and facilitating better interventions to eliminate inequities in healthcare. For example, based on the evaluation of ingredients in Fig 7, we observed that among different types of long-acting insulins prescribed to non-Hispanic White patients, the more expensive type, insulin detemir, had a higher prescription rate.

## Discussion

The two approaches, logistic regression and equity-metric based visualization, evaluate utilization disparity in healthcare access from different perspectives. The logistic regression model is used to compare access and utilization to a reference group while controlling for potential confounders. It can consider the potential effects of drug treatment shifts caused by official guidelines and new knowledge on drugs from new studies, such as cardiovascular outcome trials [38], over time. Medications that entered into the U.S. market earlier may lead to a higher prescribing rate. The equity-metric based visualization approach helps identify whether subgroups have adequate access and utilization of healthcare in relation to the specified target population, effectively addressing the bias from unbalanced subgroup proportions. But the equity approach can only address a limited number of discretized control factors. However, as the number of factors of interest increases, more data is required to adequately represent the subgroups, and the effectiveness of the visualizations decreases. In general, the logistic regression model focuses on the sameness across people irrespective of being rational or not; our fairness-based visualization approach focuses on treating populations fairly but differently rather than assuming sameness across people.

Our results revealed disparities in access to health care services, including vaccines and antidiabetic medications, based on demographic and socioeconomic factors. For instance, the access to certain classes of antidiabetic medications, particularly the newer ones, were associated with race/ethnicity and insurance status. These identified disparities could provide guidance to the health care providers on reducing existing inequities.

Our results also revealed the importance of multivariate analysis conditioned on disease-specific health conditions such as HbA1c levels of patients to evaluate access to healthcare across subgroups defined over demographic and socioeconomic attributes that should not influence health outcomes. For example, inadequate access to DPP4-Is in the Hispanic community was only observed when they had non-private insurance. However, some disparities could be explained by disease-specific or treatment-specific guidelines provided by health agencies such as the CDC. For example, disparities in HPV vaccination rates associated with age are likely a result of CDC recommendations. The CDC recommends HPV vaccination for people up to 26 years old, but some individuals over 26 may still receive it if not adequately vaccinated when younger [39]. Additionally, collaboration with physicians and endocrinologists is necessary to uncover clinically relevant findings and gain a deeper understanding of the associations between disease-specific factors, treatments, and disparities. For example, the disparity of receiving 2-dose or 3-dose HPV vaccines depends on the time between a patient’s first and second HPV vaccinations, which should not be interpreted as a potential inequity in health services utilization.

Our experiment had some limitations. First, the self-reported prescription medication information by participants may be subject to reporting bias. The degree of disparities identified can deviate from the true one in the target population if the self-reported data is significantly different from the real medication use. Second, some medication/ingredient class samples were not large enough in the subgroup analyses to provide further exploration. Furthermore, since NHANES does not provide all the health conditions required to calculate CCI, which is associated with prescribing decisions of antidiabetic drugs, it is important to examine more comprehensive data resources to obtain a more accurate understanding.

With the use of the semantic infrastructure, it is possible to mitigate some of the limitations, such as further expanding the drug selection criteria, since the NHANES Knowledge Graph expands the original data to include content from existing databases such as ICD10-CM [40] and RxNorm [41]. However, we have not leveraged these expansions in our current analysis since our original goal was to be able to compare the results of analysis generated from datasets manually derived from raw NHANES data against analysis from datasets derived from our semantic infrastructure.

Further research should include the study of access and utilization of other healthcare services, such as hospitalization and COVID-19 vaccination, while considering a comprehensive list of socioeconomic and demographic characteristics that may influence health status and outcomes. Additionally, the development of a semantic-based evaluation framework that automatically summarizes and explains findings to physicians and policymakers would support public health and clinical decision making.

## Conclusion

Our findings provide evidence of inequitable accessibility and utilization of hyperglycemic medications and CDC-recommended vaccines, influenced by demographic and socioeconomic characteristics (e.g., race/ethnicity, poverty level, insurance type, and education level.) However, determinants of access to different healthcare are not the same, requiring disease-level analyses. These discoveries indicate the need and potential interventions to reduce preventable disparities in health care access and utilization among different populations and thus allow every person to live healthier lives.

The equity-analysis methodology we developed is powerful and can be generalized to investigate disparities in other types of healthcare access, including various prescription drugs and hospital services. The proposed equity-focused methodology effectively identifies potential determinants of access to healthcare services and impacted subgroups. Our semantic infrastructure supports the data preparation steps and facilitates the process by allowing the selection of variables based on their semantic meaning, streamlining the analysis. In place of combining and normalizing variables from several datasets across the NHANES cycles used in this analysis, this process was facilitated by user interfaces and the NHANES ontology. By visually browsing ontology terms, we could select sets of variables of interest based on their semantic meaning (e.g., “insurance coverage”), instead of manually combining several variables (such as seven variables that characterize insurance coverage). As we expand the NHANES ontology to cover additional datasets and cycles, this approach can be utilized in new studies that use NHANES data, enhancing the usefulness of the method.
